# Biomimetic Neuromorphic Sensory System via Electrolyte Gated Transistors

**DOI:** 10.3390/s24154915

**Published:** 2024-07-29

**Authors:** Sheng Li, Lin Gao, Changjian Liu, Haihong Guo, Junsheng Yu

**Affiliations:** State Key Laboratory of Electronic Thin Films and Integrated Devices, School of Optoelectronic Science and Engineering, University of Electronic Science and Technology of China (UESTC), Chengdu 610054, China

**Keywords:** neuromorphic sensory system, electrolyte-gated transistors, integrated technologies, artificial neural networks

## Abstract

Biomimetic neuromorphic sensing systems, inspired by the structure and function of biological neural networks, represent a major advancement in the field of sensing technology and artificial intelligence. This review paper focuses on the development and application of electrolyte gated transistors (EGTs) as the core components (synapses and neuros) of these neuromorphic systems. EGTs offer unique advantages, including low operating voltage, high transconductance, and biocompatibility, making them ideal for integrating with sensors, interfacing with biological tissues, and mimicking neural processes. Major advances in the use of EGTs for neuromorphic sensory applications such as tactile sensors, visual neuromorphic systems, chemical neuromorphic systems, and multimode neuromorphic systems are carefully discussed. Furthermore, the challenges and future directions of the field are explored, highlighting the potential of EGT-based biomimetic systems to revolutionize neuromorphic prosthetics, robotics, and human–machine interfaces. Through a comprehensive analysis of the latest research, this review is intended to provide a detailed understanding of the current status and future prospects of biomimetic neuromorphic sensory systems via EGT sensing and integrated technologies.

## 1. Introduction

The sensory system integrates various modalities, including visual, auditory, tactile, gustatory, and olfactory, which serve as the neural foundations for the reception and processing of sensory stimuli within the nervous system [1,2,3,4,5]. This system, consisting of sensory receptors, neural pathways, and cortical regions, governs perceptual phenomena. In humans, the complex nervous network enables bidirectional interaction between organisms and their external environment. This interaction imparts remarkable efficiency and intelligence in sensing, processing, and responding to stimuli [6,7,8,9]. Engineers and scientists have long been devoted to the investigation and development of bionic neuromorphic architectures and intelligent neuromorphic systems that emulate the functionality of biological systems [10,11,12]. Classical computing systems based on the von Neumann architecture, operating on software simulations of biological neural networks, have traditionally utilized centralized, sequential processing and a store-and-compute separation manner [13]. However, biological neural systems inherently adopt a distributed, parallel, and event-driven approach, processing information through synapses and neurons [13,14]. In comparison, the latter are significantly more compact and efficient when handling complex real-world scenarios [15]. Consequently, investigating the complex cognitive aspects of information interaction and processing mechanisms in biological neural systems, especially at the neuromorphic device level, remains a paramount research focus in the field.

To create artificial neuromorphic devices and systems capable of performing tasks with human-like efficiency and intelligence, initial efforts utilized traditional complementary metal oxide semiconductor (CMOS) technology to emulate various brain-like functions [16,17,18]. However, due to intrinsic limitations in CMOS technology, such as integration density and power consumption, which increasingly fail to meet performance and energy efficiency requirements, the focus has shifted towards novel electronics that better mimic neuro functions [19,20]. Among these, two-terminal devices, including memristor [21,22,23,24], phase-change memory (PCM) [25,26,27,28], and magnetic tunnel junctions (MTJ) [29,30,31,32,33], have been extensively explored and implemented in neuromorphic designs. Despite addressing several challenges, some issues for bioinspired neuromorphic applications remain unresolved. For instance, the stochastic nature of crystallization and device variability in PCM can hinder precise parameter control, introduce noise, and complicate the development of efficient algorithms, necessitating additional circuitry [27,34]. MTJ and memristors offer significant advantages for neuromorphic computing due to their nonvolatility and energy efficiency [35]. However, they still face substantial challenges related to scaling, performance variability, and integration with existing technologies [36,37]. These issues underscore the necessity for further optimization in device mechanisms and fabrication processes. As a consequence, tri-terminal-based transistors have emerged as promising candidates for advancing neuromorphic device development [13,14,17,38,39,40]. The inherent capacity of transistors to be effectively modulated via manipulation of the gate electrode bears resemblance to biological processes, thus garnering significant attention for their integration into neuromorphic systems [41,42,43]. Notably, electrolyte-gated transistors (EGTs), reliant on mixed ionic-electronic coupling and transport mechanisms, offer biocompatibility [14,44], low operating voltage [42,45,46], reliable mechanical flexibility [6,47,48], and simple fabrication processes [7,46]. Such attributes have facilitated the emulation of biological neural network behavior, enabling the realization of sophisticated computational architectures and the exploration of pioneering bioinspired neuromorphic applications. However, the majority of current reported reviews on EGT primarily focus on the design of p-type and/or n-type materials and device structures, the detection of specific biomarkers, and the study of synaptic electronics [25,49,50,51]. Overviews related to the construction of synapse, neuro, and more complicated bioinspired neural systems are still lacking.

In this review, we present a meticulous analysis of recent advancements and prospective avenues within the domain of bio-inspired electronics utilizing electrolyte gated transistors. Initially, we expound upon the foundational principles underlying the utilization of electrolyte gate transistors for the realization of neuromorphic devices, encompassing synaptic and neuros functionalities. Subsequently, we furnish an exhaustive exposition on bio-inspired neuromorphic electronics, with a particular emphasis on the evolution of pioneering bionic systems endowed with sensing, information processing, and cognitive modulation capabilities. Furthermore, we delineate advancements in interactive systems characterized by perceptual, storage, and computational functionalities, notably encompassing tactile, visual, chemical, and other multimode neuromorphic systems. Finally, we conclude with a perspective on development and research focal points, alongside the challenges associated with translating these innovations into practical applications.

## 2. Fundamentals of Nervous Sensory System

Biomimetic nervous sensory systems represent a fascinating fusion of biology, engineering, and materials science that aims to replicate the sensory capabilities of living organisms in artificial systems. This interdisciplinary field draws inspiration from the complex and efficient ways in which natural sensory systems, such as humans and animals, detect and respond to environmental stimuli [17,52]. Creating advanced sensors and robotic systems with enhanced functionality and adaptability by mimicking these biological systems is at the core of the creation of biomimetic neuromorphic systems [17,52].

**Figure 1:**
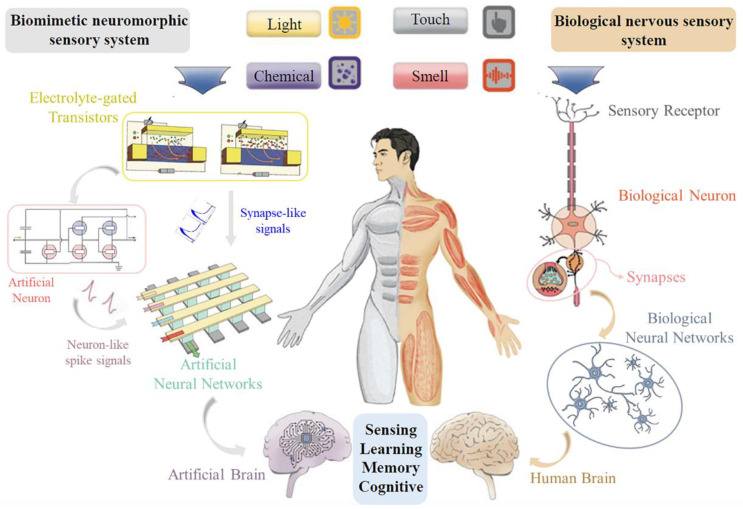
Schematic diagram of the integral architecture of the biomimetic neuromorphic sensory system and the biological nervous sensory system, depicting, on the left, the pathway from sensing to the neurological signal acquisition element to the implementation of the artificial brain, and on the right, the signaling process in a living organism [76,77].

Generally, the human nervous system, composed of the central nervous system (CNS) and the peripheral nervous system (PNS), is integral to receiving, processing, and responding to external stimuli [6,12,53]. In particular, the PNS connects receptors, muscles, and glands, serving as a conduit for transmitting nerve impulses and eliciting muscle responses [54]. In addition, the CNS, which is composed of approximately 10^11^ neurons and 10^15^ synapses within the cerebral cortex and spinal cord, aggregates impulse signals from the PNS to form sensations, integrating sensory information for higher cognitive functions such as recognition, imagination, and reasoning [19,55,56,57]. As shown in Figure 1, when sensory nerve cells receive external stimuli, such as light, pressure, odor or taste, electrical signals are generated and propagate along the cell membrane [58,59,60]. These electrical signals are converted into chemical signals at the axon terminal. Neurotransmitters then diffuse across the synaptic gap to the postsynaptic membrane, binding to receptors and triggering a potential change in the postsynaptic membrane. This process facilitates the rapid transmission of nerve impulses between neurons [61,62]. This stimulus-induced signal travels through multiple synapses and neurons in the neural network, which undergoes a modulation of synaptic weights and the integration of neurons; the resultant sensory signals are conveyed to the CNS, culminating in the formation of sensations and perceptions in the cerebral cortex [1,41,63]. This parallel signal-processing approach provides a more efficient and less energy-intensive solution than current digital computers [15,55,64,65]. Given these advantages, the development of biomimetic neuromorphic devices or systems to realize bio-like parallel computing is imminent. Similarly, in an artificial nervous system, environmental changes such as light, pressure, and chemicals are transduced into voltage or current pulses by sensing elements [66,67,68,69,70]. These electrical signals can be directly transmitted to artificial neural networks in the form of synapse-like signals for computation [68,69,70] or converted into neuron-like spikes via artificial neuron devices [71,72,73]. The artificial neural network emulates the functionality of a biological neural network, processing and analyzing signals to develop artificial perception and cognitive abilities, thereby creating a hardware-based artificial intelligence system [74,75]. Therefore, the design and implementation of synapses and neurons with biologically-like functions provides a solid foundation for the construction of the entire biomimetic system.

### 2.1. Electrolyte-Gated Neuromorphic Transistors

EGTs are suitable for neuromorphic devices due to their ability to mimic the ion-based signaling in biological neurons [40,49]. The electrolyte–semiconductor interface allows low-voltage operations and high capacitance, enabling efficient ionic–electronic coupling transport and the modulation of channel conductivity [46,78,79]. This facilitates the emulation of synaptic plasticity, which is crucial for learning and memory functions in neuromorphic systems. Additionally, EGTs can be fabricated using flexible, biocompatible materials [69,80,81], making them ideal for integrating with biological tissues [82,83,84]. Their tunable response [85,86] and compatibility with various substrates [80,87,88] further enhance their potential for developing advanced, adaptive neuromorphic circuits.

### 2.2. Electrolyte-Gated Transistor Synapse

**Figure 2:**
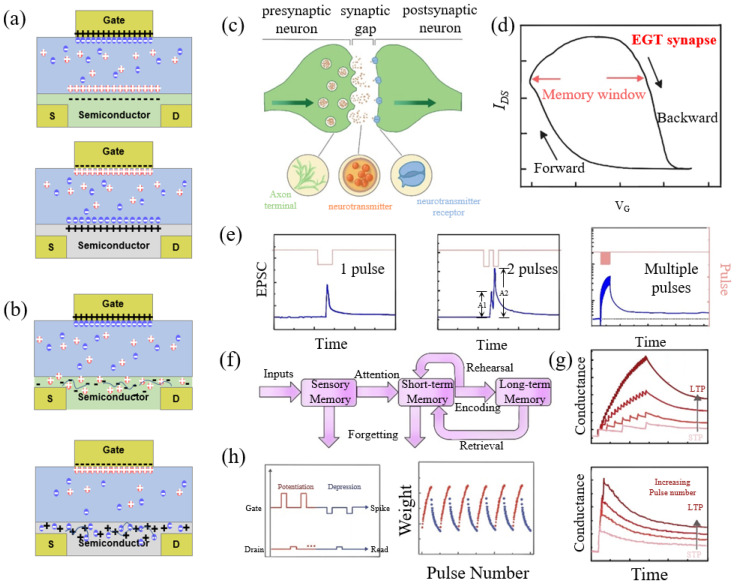
Working mechanisms of the operation of (**a**) EDL transistors and (**b**) ECTs at different gate voltages [75]. (**c**) Schematic diagram of a neurotransmitter-mediated chemical synapse between biological neurons. (**d**,**e**) Schematic illustration of typical synaptic transistor characteristics [46,92]. (Pink lines represents the pre-synaptic stimulis, and blue plot indecates the EPSC) (**f**) Three major stages of memory and forgetting in the human brain, as described by the Atkinson–Shiffrin memory model [93]. (**g**) Transformation from STP to LTP by stimulation with different frequencies and pulses [94]. (**h**) Artificial synaptic learning and forgetting processes [95]. (Red dots indicate increasing synaptic weights during potentiation corresponding to the left panel, and blue dots shows decreasing synaptic weights during depression).

EGTs are three-terminal devices analogous to field-effect transistors (FETs), wherein amplification is achieved by modulating the conductivity of the semiconductor layer through ion migration [42,45,46]. An ionically conducting and electronically insulating electrolyte serves as the gate insulator [45,46]. Based on the permeability of the semiconductor material to ions, EGTs operate through two fundamental mechanisms. The first mechanism involves electric double layers (EDLs), where ions from the electrolyte cannot permeate the semiconductor (Figure 2a). When a gate voltage is applied, electrolyte ions of the opposite polarity migrate toward the gate electrode–electrolyte interface to balance the charge in the gate electrode, while another ion migrates to the electrolyte–semiconductor interface. To maintain the EDL at the electrolyte–semiconductor interface, carriers within the semiconductor accumulate (or deplete) at the interface, functioning as nanoscale capacitors [45,89]. The thin nature of these capacitors enables very high capacitance values and substantial electric fields (10^6^ V/cm) [45,90], facilitating efficient carrier modulation and variation in semiconductor layer conduction. The second mechanism involves electrochemical transistors (ECTs), which are based on ion-permeable semiconductors. The detailed working mechanism is illustrated in Figure 2b, for both p- and n-type semiconductors. During the operation of an ECT, the gate voltage drives ions to inject/eject from the transistor channel, altering the doping state and conductivity of the active layer. Under the bias of the drain-source voltage, the resulting electronic current varies significantly [79,91]. Therefore, the current response of the EGT can be modulated by the material to achieve the desired output characteristics for synapse or neuro applications.

In biological systems, the synapse serves as the connecting interface between two neurons and functions as a storage unit for learning and memory. Biological synapses are categorized as electrical or chemical [96,97]. Electrical synapses, which rapidly transmit bidirectional signals, are predominantly found in invertebrates. In contrast, chemical synapses utilize neurotransmitters to transport neural signals across synaptic clefts between neurons. As illustrated in Figure 2c, upon the arrival of an action potential at the presynaptic membrane, neurotransmitter-filled synaptic vesicles are released into the synaptic cleft through membrane fusion. These neurotransmitters diffuse to the postsynaptic membrane, where they bind to specific receptors, opening sodium or potassium ion channels and generating action potentials in the postsynaptic neuron [97]. This process allows more complex signal transmission than electrical synapses and enables precise synaptic strength control during learning and memory processes. Consequently, the synapses in the human brain are primarily chemical synapses, which facilitate more complex functions [39,97]. Developing devices to emulate chemical synaptic functions is a key focus in artificial synapse research.

Analogously to synapses, EGTs can modulate current signaling via ion transport [38,49,98]. In this context, the gate electrode can be considered analogous to the presynaptic membrane and the drain electrode to the postsynaptic membrane, with the drain current responding to stimulation by the gate voltage [99,100]. Upon the removal of the gate voltage, the electrolyte ions that form the electric double layer or are doped into the semiconductor slowly diffuse back to their initial state, resulting in a relaxation time before the drain current fully recovers. Due to the energy barrier for ion migration and redox reactions, the mobilized ions remain relatively stable within the channel or electrolyte, leading to nonvolatile conductance modulation [42,46,101,102]. Consequently, significant hysteresis is typically observed during I_DS_-V_G_ sweeps (Figure 2d), with the memory window (MW) defined as the maximum separation of the voltages when the drain current is identical [38,103].

In the nervous system, excitatory receptors induce excitatory post-synaptic currents (EPSC) (Figure 2e), bringing the postsynaptic neuron closer to the threshold for action potential [104]. Conversely, inhibitory receptors result in inhibitory post-synaptic currents (IPSC), driving the postsynaptic neuron further from the action potential threshold [105,106]. Synaptic plasticity refers to changes in communication strength based on the synapse’s activity history [107,108]. Paired-pulse facilitation (PPF), a critical form of short-term plasticity (STP), can be characterized by an increased post-synaptic current (PSC) amplitude triggered by two pulses with a second spike (A2) compared to the first spike (A1), particularly when the second spike follows the first in quick succession [109,110,111]. The facilitation ratio (A2/A1) depends on the interval between spikes and can be represented by a double exponential decay function [109,112]. Pulsing through multiple gate voltages also excites stronger drain currents to mimic the process of gradual learning at neural synapses [99,113,114,115].

In the human learning process (Figure 2f), electrical signals from external stimuli produce STP, and continuous or repeated stimuli can induce long-term plasticity (LTP). Neuromorphic EGTs can be converted from STP to LTP characteristics by adjusting the pulse width and number of pulses, enabling a more sustained conduction state (Figure 2g). Furthermore, constant repetitive potentiation increases the channel conductance, whereas constant repetitive inhibition has the opposite effect, leading to the continuous updating of synaptic weights (Figure 2h). This mechanism underpins the realization of learning and memory functions [116,117]. In summary, a wide range of biomimetic functions are achieved through electrical signal modulation. Efficient, linear characterization remains a challenge that must be addressed to fully realize the potential of neuromorphic electronics for brain-like computation and learning [118,119].

### 2.3. Electrolyte-Gated Transistor Neurons

**Figure 3:**
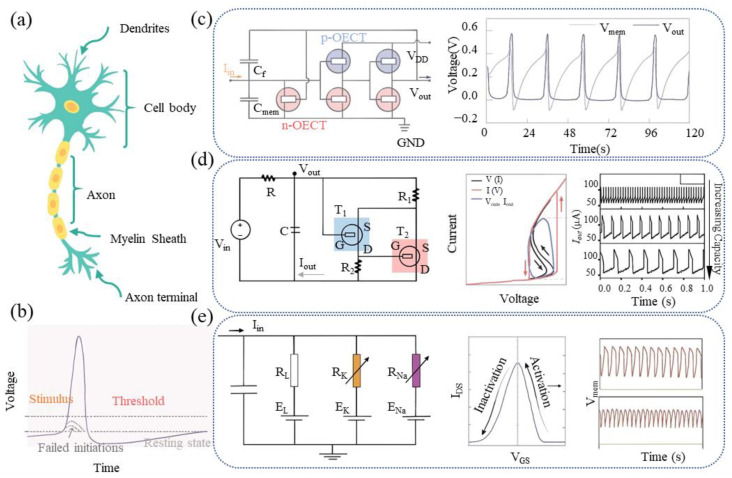
(**a**) Structure of biological neurons and (**b**) bioelectric signals generated by neurons. (**c**–**e**) Various model-based artificial neural components and their corresponding neural pulse signals [73,130,131,132,133].

Neurons are fundamental components of the nervous system, crucial for sensing stimuli and transmitting excitation. In the resting state, the cell membrane is highly permeable to K^+^, leading to K^+^ efflux and maintaining a negative membrane potential, as shown in Figure 3a,b [120]. During nerve excitation, Na^+^ channels open, allowing Na^+^ to enter the cell. This influx of Na^+^ triggers positive feedback, further enhancing Na^+^ entry and raising the membrane potential to generate an action potential. When the cell potential reaches a threshold, the membrane potential gradually returns to the resting state as the Na^+^ channels close [121,122,123].

There are two primary models for simulating nerve impulse activity. The Hodgkin– Huxley model describes various ionic movements within neurons [124,125,126]. Alternatively, mathematical models such as the integrate-and-fire (IF) and the leaky integrate-and-fire (LIF) models describe changes in membrane potentials without considering ion diffusion properties [127,128]. The IF model proposes that the membrane potential increases upon receiving external stimuli until it reaches a threshold, generating an action potential. In contrast, the LIF model accounts for the spontaneous decrease in membrane potential due to ionic leakage between stimuli, aligning more closely with biological realities, and it is commonly used for artificial neurons [129].

To physically simulate LIF behavior, various circuits based on EGTs have been proposed. As shown in Figure 3c, the axon hillock circuit was employed to construct a LIF-type pulsed organic electrochemical neuron (OECN) [130,131]. In this model, the capacitor C_mem_ accumulates the input current and generates an output voltage (V_out_) through an in-phase amplifier circuit and a positive feedback capacitor (C_f_). This output activates T_reset_, returning the artificial neuron to its resting state, and producing the voltage spike. Figure 3d also demonstrates a cascade configuration using two organic electrochemical transistors (OECTs) to create two competing current pathways [73,132]. When the input voltage exceeds the threshold, the gate voltage (V_G_) of T_1_ rises as the capacitor charges, eventually turning off T1 and decreasing the V_G_ of T_2_, allowing current to flow through T_2_. This switching of current pathways causes a sudden decrease in resistance, achieving negative differential resistance. This process produces a spontaneous oscillatory current and voltage output, mimicking the neuron’s spiking sequence during excitation. When the input voltage is below the threshold, the OECT does not trigger oscillatory behavior.

Additionally, two OECTs driven by different ions can be used to construct artificial neurons [133]. The Na^+^ OECT exhibits an antiambipolar nature, as illustrated in Figure 3e, while the K^+^ OECT uses thicker channels for greater conductivity and larger parasitic capacitance, resulting in slower turn-on times. This configuration successfully simulates the Na^+^ and K^+^ permeability during biological nerve impulses, mimicking properties such as tonic spiking and secondary ion control.

## 3. Application of Biomimetic Neuromorphic Sensory Systems

Receptors, neurons, and synapses in biomimetic systems collaborate efficiently to recognize and process complex sensory information. Physiologically, human receptors detect various stimuli that propagate along axons and transmit through synapses to postsynaptic neurons, enabling further processing and the recognition of the corresponding neural information. Biological systems feature diverse receptors specialized in detecting specific stimuli, such as touch [3], taste [4,134], smell [2], vision [5], and hearing [54]. This chapter primarily summarizes bio-inspired neuromorphic sensory systems activated by artificial bionic receptors, including tactile neuromorphic systems, visual neuromorphic systems, chemical neuromorphic systems, and multimode neuromorphic systems.

### 3.1. Tactile System

**Figure 4:**
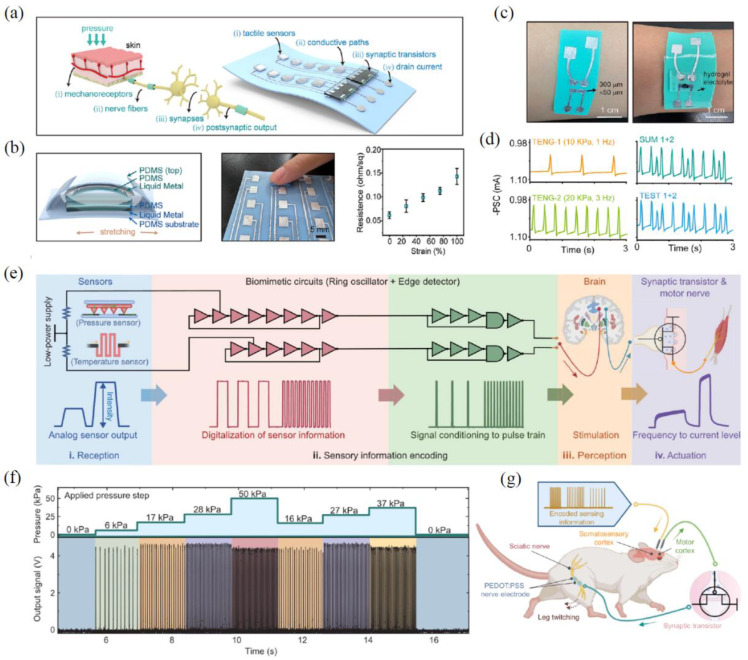
Tactile perception system based on EGTs. (**a**) Biological and neuromorphic mechanisms for tactile sensing systems, with (**b**) TENG for mechanosensory and power supply functions. (**c**,**d**) Optical image of the integrated tactile system showing two TENG inputs and their synaptic signals [134]. (**e**) Schematic diagram of the overall process for implementing an artificial sensory (tactile and temperature) motion loop, combining a ring oscillator and an edge detection module. (**f**,**g**) Structural diagram of the artificial sensorimotor system and pulse train output under five variable stages, according to the pressure sensor [135].

In the somatosensory system, the perception, transmission, and processing of tactile information rely on distributed parallel neural networks, effectively addressing complex and unstructured real-world problems. These electrical signals travel along nerve fibers to the presynaptic membrane, inducing the release of neurotransmitters and causing post-synaptic membrane oscillations, ultimately conveying tactile sensations to the brain, as illustrated in Figure 4a,b. Wu et al. [134] demonstrated a skin-conformable neuromorphic tactile system comprising TENG tactile sensors (also functioning as a self-powered block) and hydrogel-gated OECT synapses. This system exhibits pressure-sensing, synaptic, and mechanical characteristics similar to those of the epidermis, including ultralow-sensitive detection with a linear sensitivity of 0.04 kPa^−1^ within the range of 0.24 to 23.56 kPa, numerous memory states, symmetric weight updates, and excellent stretchability (Figure 4c). Such a neuromorphic tactile system is capable of sensing, integrating, and distinguishing multiple pressure inputs, as evidenced by demonstrations of a Morse code reader and a handwritten alphabet-based human–machine interface (Figure 4d). Although current tactile neuromorphic electronics have achieved skin-like softness and the ability to mimic peripheral nervous system functions, with applications in prosthetics and robotics, these devices still need to emulate the signal patterns of biological nerve impulses (like neuros). This emulation is essential to achieve natural tactile sensing and to encode tactile input through frequency-modulated signals, thereby regulating corresponding motor activities. Therefore, Wang et al. [135] have developed a haptic neuromorphic system for a monolithic soft prosthesis through strategic design and engineering modifications to material properties, device structure, and system architecture. This system is capable of multimodal sensing, neuromorphic pulse sequence signal generation, and closed-loop actuation (Figure 4e). By further integrating transistors, the authors developed a complex neural circuit capable of directly modulating pressure-sensing signals into variable frequencies. The sensor collects external stimuli, while the ring oscillator generates action-potential pulse sequences (see Figure 4f for pressure modulation effects). This achieves a sensory system that simulates the tactile coding pulses of biological neural receptors. Furthermore, with the solid-state synaptic transistor inducing stronger actuation in response to increasing pressure stimuli, such a neuromorphic system can connect to a rat’s somatosensory cortex to mimic the skin’s feedback response under tactile-triggered movement in the cortex (Figure 4g). The evoked motion signals then stimulate the sciatic nerve via artificial synapses to activate downstream muscles, resulting in an artificial sensorimotor loop. In general, the synaptic transistor-based building blocks of tactile neuromorphic systems are also under continuous development, gradually shifting from synaptic electronics to the building of pulsed neurons in the integrated closed-loop system of the nervous system.

### 3.2. Visual System

**Figure 5:**
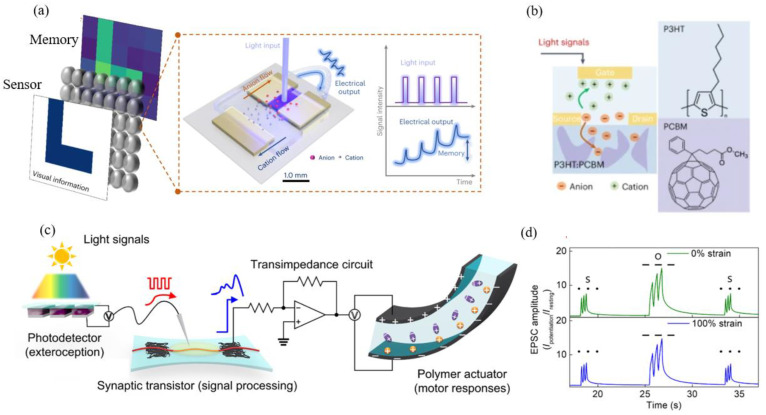
(**a**) Biological optical synapses and artificial photoelectric synapses. (**b**) Photoresponsive principles and photoactive materials for EGTs [136]. (**c**) Photomodulated neuromorphic systems, comprising photodetector units, neuromorphic transistors, amplification circuits, and actuators. The red arrow indicates the inputs to the synaptic transistor, and the blue arrows represent the synapse-like output. (**d**) Neuromorphic signaling of visible light response based on various encoding modalities [145].

Beyond developing bio-electronic devices or humanoid robots with advanced tactile neuromorphic system functions for the future, exploring light-responsive, optically gated synaptic transistors (organic photonic synapses) offers opportunities to simulate the image preprocessing and recognition functions of the human retina [136,137,138,139]. To date, FETs have been among the primary platforms for organic photonic synapses [140,141]. However, due to the charge shielding effect at the channel–dielectric interface, FETs still face intrinsic obstacles in achieving linear multilevel conductance states, which hinders their application in neuromorphic computing [140]. Therefore, Chen et al. [142] have proposed an artificial retina consisting of an array of optoelectronic synaptic devices, each of which has the combined functions of light perception, optoelectronic signal transduction, and memory (Figure 5a) The formation of memory in the human brain relies on ion-flux-driven synaptic transistor activity. As a result, the artificial retina is capable of efficiently sensing, remembering, and recognizing image information. More notably, to simulate this ion-flux-modulated synaptic activity and construct light-regulated photoelectrical synapses, the building blocks of the artificial retina incorporated a conventional photoactive layer (poly(3-hexylthiophene) (P3HT) and [6,6]-phenyl-C61-butyric acid methyl ester (PCBM)) into the transistor channel. In this context, light absorption can interfere with electrochemical doping, as light-induced charge carriers in bulk heterojunctions facilitate charge compensation through ion transport from the electrolyte to the channel. Consequently, light can serve as a presynaptic input to generate a postsynaptic electrical signal (Figure 5b). While organic optoelectronic artificial synapses present a feasible approach for developing vision-inspired neuro-electronics by emulating biological synaptic functions, research in the domain of electrochemical transistors has been sparse, predominantly focusing on material exploration and the development of individual devices to mimic vision-related synaptic responses and memory characteristics [143,144]. In an attempt to achieve a more integrated visual neuromorphic system, Lee et al. [145] proposed an organic optoelectronic sensing motor system based on the optogenetic principle. This system uses organic photoelectronic synapses and a neuromuscular system based on stretchable organic nanowire synaptic transistors (s-ONWSTs) (Figure 5c). Voltage pulses from a self-powered photodetector triggered by an optical signal drive the s-ONWST, and the resulting informative synaptic output can be used not only for optical wireless communication in human–machine interfaces, but also for the optically interacting actuation of artificial muscle actuators, in the same way as biological muscle contractions. Furthermore, the visual neuromorphic system is also soft and maintains output stability, even when stretched (Figure 5d). As electrolyte-gated transistor technology advances, vision neuromorphic systems are expected to play a crucial role in various fields, leading to smarter, more efficient, and more adaptive systems capable of performing complex visual tasks in real-world scenarios.

### 3.3. Chemical System

**Figure 6:**
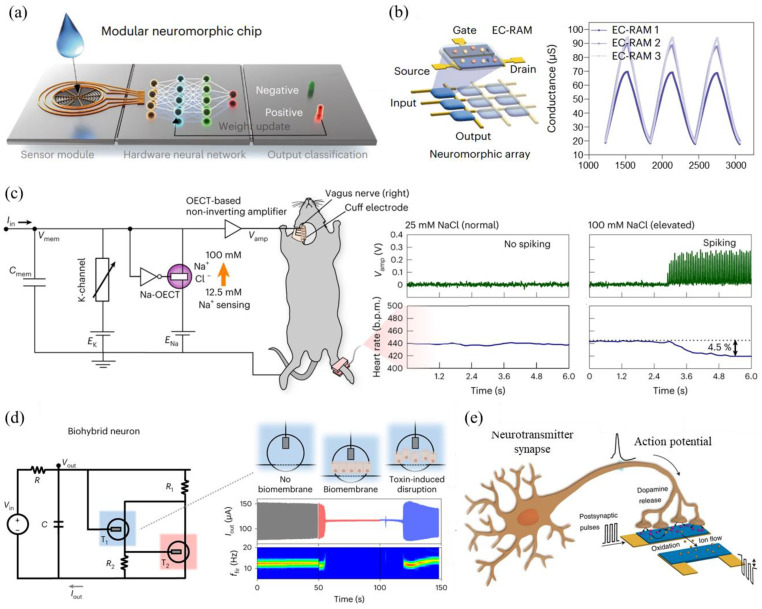
(**a**) A schematic of a modular neuromorphic biosensor showcasing various functions. (**b**) Conductance values of electrochemical random-access memories within a small neuromorphic array, along with three electrochemical random-access memory devices serving as synaptic weights [146]. (**c**) The c-OECN circuit of Na-OECT for Na^+^ ion sensing and its integration with the mouse vagus nerve using an OECT-based amplifier. The image also displays the amplifier output and corresponding heart rate changes at various concentrations of NaCl [132]. (**d**) A schematic representation of a biohybrid neuron, composed of an organic artificial spiking neuron and a doped biofilm. The image further illustrates the variation of I_out_ of biohybrid neurons over time and corresponding time-frequency analysis [72]. (**e**) A schematic representation of an artificial neuron in conjunction with a biological neuron, where the dopamine concentration at the cell–device interface is contingent on the firing rate of the presynaptic neuron. Moreover, the change in the postsynaptic current is dependent on the pulse rate of the presynaptic and postsynaptic salient regions, giving rise to the associated spike-learning mechanism [147].

Neurons could signal through membrane potential peaks that are determined by ion channel voltages, as well as ion- and neurotransmitter-dependent conductance across the cell membrane. Potassium ions, sodium ions, and other ions pass through ion channels on the surface of the cell membrane to change the membrane voltage, thereby generating an action potential [122,123]. Therefore, the simulation of ions in and out of ion channels by electrolyte-gated transistors is an important component in the realization of a bionic neuromorphic system. However, leaky integrate-and-fire model neurons based on inorganic semiconductors do not include complex ion channel dynamics and can only simulate a limited number of neural features [127,128]. OECTs based on ion–electron hybrid conducting polymers are attractive in this regard due to their biocompatibility, biologically compatible switching speeds, low operating voltages, and ion–electron coupled transport properties that can be modulated by external ions or molecules. Van de Burgt et al. [146] prepared ion-selective organic electrochemical transistors (IS-OECTs) using modified treated PEDOT:PSS to monitor physiological levels of potassium and chloride ions without the use of ion-selective membranes and constructed modular neuromorphic biosensors based on this IS-OECT (Figure 6a). The IS-OECT serves as an electrochemical stochastic memory, where differences in conductance values due to changes in ion concentration are used as synaptic weights. It is trained by a hardware-based neural network that uses error-signaling feedback to modulate the conductance of an organic neuromorphic device, enabling accurate disease classification (Figure 6b). Simone et al. [132] demonstrated a BBL-based ion-tunable anti-ambipolarity to mimic the activation and inactivation of sodium channels in biological neurons, leading to conductance-based organic electrochemical neurons (c-OECN). By connecting the c-OECN to the vagus nerve, Na-OECT can detect the Na^+^ concentration, produce spikes of intensity corresponding to it, and electrically activate the vagus nerve, which causes changes in the heart rates of mice (Figure 6c). This demonstrates that it is possible to respond to specific concentrations of biochemical signals via c-OECN, leading to event-based sensing, biointegration, and neural activation functions. Paschalis and colleagues [72] report an organic artificial spiking neuron consisting of only two OECTs (T_1_: depletion transistor and T_2_: enhancement transistor) that modulates neuronal excitability and triggers spiking by altering ,in situ, the concentration of biomaterials (e.g., ions or dopamine) in the fluid [73]. An epithelial cell membrane was also incorporated between the T_1_ gate and the channel to create a biomixed interface that modulates the spiking properties of the artificial neuron in real time through the cell membrane barrier. Thus, this organic artificial spiking neuron is capable of local and in situ neuromorphic sensing and biological interfaces (Figure 6d). Francesca et al. [147] constructed a neuromorphic device that realizes the basic functions of biological neurons through a neurotransmitter-mediated neuromorphic device (Figure 6e). The authors used PC-12 cells as dopamine synaptic precursors and an organic neuromorphic device as a postsynaptic body, which combined to form functional biohybrid synapses. The dopamine released by PC-12 cells can be locally oxidized at postsynaptic terminals by gate electrodes, leading to changes in the conductivity of active layer channels at postsynaptic terminals, thus producing transient, short-term, and long-term enhancement analogous to that in biological neural networks.

### 3.4. Multimodal System

**Figure 7:**
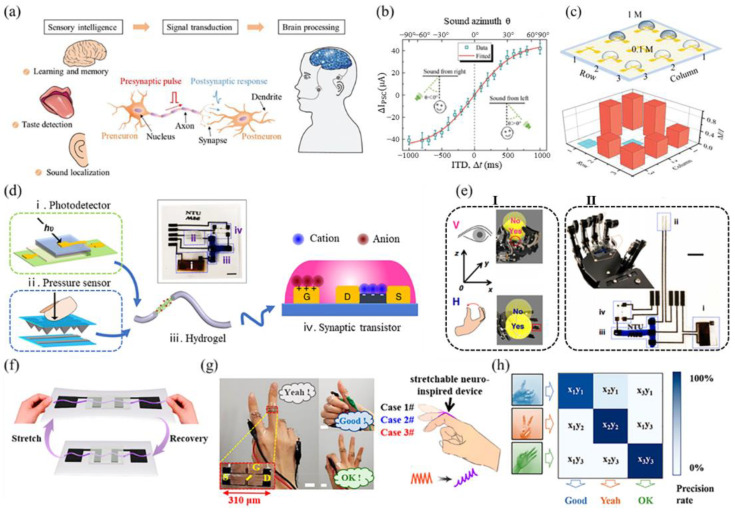
(**a**) Signal processing in biosensory systems, in which presynaptic impulses from a preneuron are transmitted through the axon to the synapse to elicit a postsynaptic response in response to an external stimulus. (**b**) The direction of the sound is determined by calculating the difference between the currents of the two post-neurons (ΔI_PSC_) as a function of time. (**c**) Detection of 0.1 M and 1 M acetic acid and generation of taste mappings in a 3 × 3 array artificial tongue arrangement scheme [148]. (**d**) BASE patches for visual and tactile fusion. Subfigures (**i**) to (**iv**) show the photodetector, pressure sensor, hydrogel (stained with 0.04% methylene blue), and synaptic transistor, respectively. The scale bar is 5 mm. (**e**) Visual–haptic fusion motion control. Subfigure (**I**): “Yes” and “No” positions inferred from visual (top, pink) or haptic (bottom, blue) feedback. The position is labeled as “yes” if the robot’s hand can hold the ball based on one type of sensory feedback, and “no” otherwise. Subfigure (**II**): Enlarged image of the modified BASE patch and the BASE patch on the robot. The scale line is 5 mm [149]. (**f**) Schematic of NISNT after repeated stretching and recovery. (**g**) Photographs of NISNT attached to the knuckle in three gestures, and schematic diagrams of the joint in three different degrees of finger bending (case #1, straight; case #2, 30°; and case #3, 45°), The scale bar is 1 cm. (**h**) The confusion matrix for three different gesture categorization tests with different exposure times and numbers of iterations [150].

The human perceptual nervous system monitors complex environments by integrating multiple sensory inputs into a single neuron, through which sensory signals are transmitted to the brain for processing. The recognition of multimodal information is achieved through the processing of excitatory postsynaptic currents generated by artificial salient transistors interacting with multimodal stimuli. Liu et al. [148] developed synaptic devices based on electrolyte-gated vertical organic field-effect transistors. These devices feature a nanometer-thick P3HT channel layer that enables the formation of an electric double layer at the electrolyte–channel interface, resulting in a large capacitance. Consequently, the power consumption for a single synaptic event is significantly lower than that of biological synapses. These synaptic transistors can emulate various synaptic mechanisms, such as paired-pulse facilitation, short-term potentiation, long-term potentiation, and long-term depression (Figure 7a). An artificial neural network composed of arrays of such synaptic transistors was able to simulate image learning, memory function, sound localization, and sour-taste detection (Figure 7b,c). Vision and touch, as fundamental perceptual functions in human beings, serve as the gateways to perceiving and understanding the world. Chen et al. [149] developed a bimodal artificial sensory neuron (BASE) utilizing hybrid ionic/electronic neuromorphic electronics for visual–tactile fusion (Figure 7d). This system transmits electrical signals from photoelectric and pressure sensors through ionic cables to synaptic transistors, converting them into transient channel currents. The functionality of BASE was merged with skeletal muscle tubes to mimic limb motor control based on visual–haptic fusion, effectively demonstrating motor control at a cellular level using bimodal sensory cues (Figure 7e). Bionic neuromorphic systems that combine visual and haptic inputs are often limited in flexibility for wearable applications. To address this limitation, Xu et al. [150] introduced a poly(3-hexylthiophene)/polyethylene oxide (P3HT/PEO) nanowire-channel intrinsically stretchable neuromorphic transistor (NISNT). This device conforms well to the skin, exhibiting excellent tensile durability even after repeated stretching (Figure 7f). The NISNT integrates synaptic and haptic functions, serving as a tactile afferent nerve and artificial synapse attached to the finger for gesture monitoring (Figure 7g). Moreover, due to the photosensitivity of P3HT/PEO, NISNT can induce postsynaptic currents when exposed to green light (at a wavelength of 550 nm and a power density of 7 μW/cm^2^). When utilized as an artificial photosensitive afferent nerve, the NISNT achieved a gesture recognition accuracy of 96.3% after iterations of the neural network algorithm (Figure 7h). Therefore, incorporating the NISNT’s visual-tactile perception fusion into artificial neural networks can significantly enhance gesture recognition accuracy.

## 4. Summary and Outlook

**Summary:** This review started with biomimicry in electronics, exhibiting that the use of biosimilar (mixed ionic–electronic coupling and transport) and low-power EGT technology to mimic the functioning of the human sensory system. This approach can improve the performance and versatility of electronic devices, thereby enabling efficient processing and adaptive learning capabilities of the human brain, forming a model for developing advanced artificial neuromorphic sensory systems. First, we provided a detailed overview of the foundational principles of using EGT for creating neuromorphic devices with synaptic and neuro functions. Additionally, we described significant progress in bio-inspired interactive neuromorphic systems with sensing and computing functions, including tactile neuromorphic systems, visual neuromorphic systems, chemical neuromorphic systems, and multimodal neuromorphic systems.

**Challenges:** Biomimetic neuromorphic sensory systems operating via EGTs also present several challenges to be addressed. One significant issue is the long-term stability and durability of EGT-based devices, as these device-based integrated neuromorphic systems must maintain consistent performance over extended periods. Second, the fabrication and integration of EGTs into larger systems also pose difficulties, requiring precise control over material properties and device architecture; power consumption is also limited, given the large output current (~1 mA), as neuromorphic systems must operate efficiently to be practical for real-world applications. Third, interfacing these devices with existing electronic systems while maintaining biocompatibility adds another layer of complexity, that of the complexity of mimicking biological processes accurately, as biological neural networks exhibit intricate dynamics that are difficult to replicate with synthetic materials. Furthermore, integrating biomimetic neuromorphic sensory systems presents several challenges: achieving accurate signal transduction and processing is complex due to the intricacies of biological sensory pathways and the need for high fidelity in mimicking sensory functions, and power consumption and computational efficiency must be optimized to handle real-time sensory data, which demands sophisticated algorithms and hardware design. Addressing these challenges requires interdisciplinary collaboration and advancements in materials science, electronics, and bioengineering to create robust, efficient, and scalable biomimetic neuromorphic sensory systems.

**Outlook:** The future of biomimetic neuromorphic sensory systems operating via electrolyte-gated transistors also holds significant promise across various domains. From the perspectives of materials and devices, continued innovation in materials science (including the synthesis of new materials, material-function modulation, the development of stretchable semiconductor materials, etc.) will probably enhance the output performance and stability of EGTs, and integration with emerging technologies (such as novel device structure, stretchable electronics, and nanofabrication techniques) could lead to more compact and efficient bio-electronic devices. From artificial and robotics perspectives, neuromorphic systems can significantly impact AI development by providing more efficient and human-like processing capabilities, and the possibility of more multimodal neuromorphics that function similarly to humans needs to be explored. Robotics could benefit from enhanced sensory systems that enable better interactions with the environment and more autonomous decision-making.
